# Molecular characterization of patients with pathologic complete response or early failure after neoadjuvant chemotherapy for locally advanced breast cancer using next generation sequencing and nCounter assay

**DOI:** 10.18632/oncotarget.4119

**Published:** 2015-05-12

**Authors:** Kyunghee Park, Moon Ki Choi, Hae Hyun Jung, In-Gu Do, Kwang Hee Lee, TaeJin Ahn, Won Ho Kil, Seok Won Kim, Jeong Eon Lee, Seok Jin Nam, Duk-Hwan Kim, Jin Seok Ahn, Young-Hyuck Im, Yeon Hee Park

**Affiliations:** ^1^ Samsung Genomic Institute, Samsung Biological Research Institute, Samsung Medical Center, Sungkyunkwan University School of Medicine, Seoul, Korea; ^2^ Division of Hematology-Oncology, Department of Medicine, Samsung Medical Center, Sungkyunkwan University School of Medicine, Seoul, Korea; ^3^ Biomedical Research Institute, Samsung Medical Center, Sungkyunkwan University School of Medicine, Seoul, Korea; ^4^ Center of Companion Diagnostics, Innovative Cancer Medicine Institute, Samsung Medical Center, Seoul, Korea; ^5^ Life Science Solutions Group, Thermo Fisher Scientific Corporation, Seoul, Korea; ^6^ Department of Surgery, Samsung Medical Center, Sungkyunkwan University School of Medicine, Seoul, Korea; ^7^ Department of Molecular Cell Biology, Samsung Biomedical Research Institute, Sungkyunkwan University School of Medicine, Suwon, Korea

**Keywords:** neoadjvant chemotherapy, pathologic complete response, breast cancer, refractory

## Abstract

Neoadjuvant chemotherapy (NAC) has the added advantage of increasing breast conservation rates with equivalent survival outcomes compared with adjuvant chemotherapy. A subset of breast cancer patients who received NAC experienced early failure (EF) during the course of therapy or within a short period after curative breast surgery. In contrast, patients with pathological complete response (pCR) were reported to have markedly favorable outcomes. This study was performed to identify actionable mutation(s) and to explain refractoriness and responsiveness to NAC. Included in this analysis were 76 patients among 397 with locally advanced breast cancer for whom a preoperative fresh-frozen paraffin-embedded tumor block was available for next-generation sequencing using AmpliSeq. The incidence of missense mutations in KRAS was much higher in patients with EF than in other groups (*p* < 0.01). In contrast, polymorphisms of the cMET gene were found in patients with pCR exclusively (*p* < 0.01).

## INTRODUCTION

Neoadjuvant chemotherapy (NAC) has the added advantage of increasing breast conservation rates with similar disease-free and overall survival compared with adjuvant chemotherapy [1, 2]. In addition, the neoadjuvant setting is a formidable research tool to unveil the mechanisms of resistance to treatment, and provides an attractive clinical setting to study the mechanisms of drug resistance *in vivo*. Triple-negative breast cancer (TNBC), which lacks expression of the estrogen receptor (ER), progesterone receptor (PgR), and human epidermal receptor 2 (HER2), is associated with a dismal prognosis despite its good response to anthracycline and taxane-based neoadjuvant chemotherapy (NAC), which yield a higher rate of pathologic complete response (pCR) [1, 3]. In particular, standard polychemotherapy results in pCR in more than 20% of patients [4, 5], and this response is considered a surrogate of increased survival compared with patients without pCR [6]. pCR to NAC indicates an extremely chemotherapy-sensitive tumor and heralds excellent long-term cancer-free survival [7]. Thus, it is currently acknowledged as a surrogate end point for therapeutic benefit, especially in HER2-positive BC and TNBC [8].

In contrast, a subset of patients who receive NAC experiences early failure (EF) during the course of therapy or within a short period after curative breast surgery. Recently, we conducted a retrospective analysis to determine the incidence and predictors of EF after NAC, with the ultimate aim of identifying patients who may not benefit from NAC. We reported that 9.6 % of the breast cancer patients who received NAC in our study developed EF within 1 year, and that the post-failure survival period of this group was shorter than that of the late-failure group [9]. Patients who had HER2+ BC or TNBC and the presence of lymphovascular invasion had a high risk of EF after controlling for other clinical and demographic variables [9]. There are no established biological predictors of EF in locally advanced breast cancer (LABC) patients who receive NAC compared with pCR.

This study was performed to identify candidate actionable mutation(s) to explain pCR and EF or refractoriness to chemotherapy in BC patient groups that may benefit to a greater extent, or not benefit from NAC.

## RESULTS

### Patient cohort

**Figure 1:**
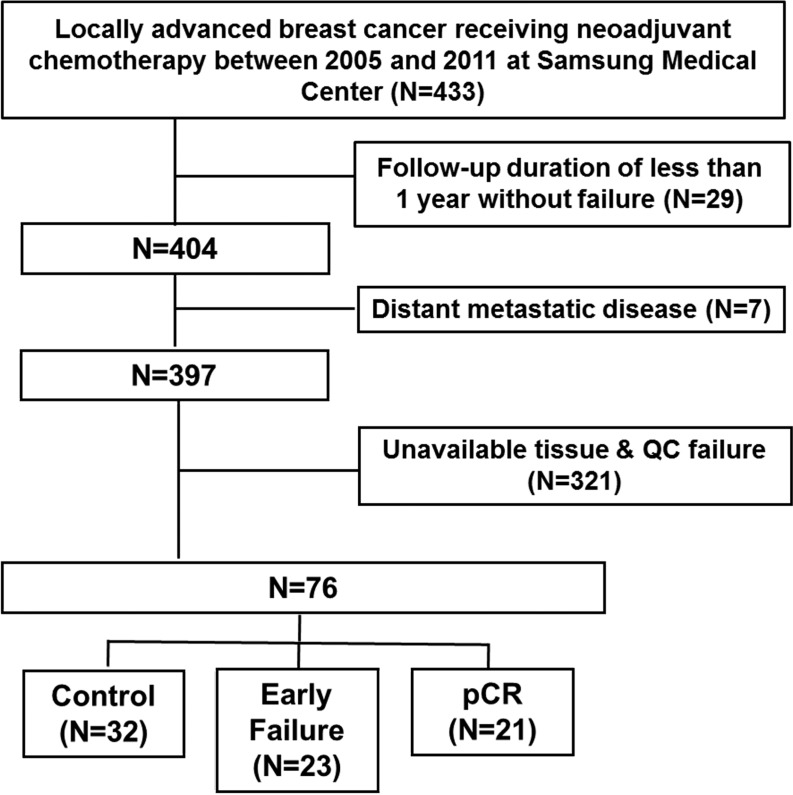
Patients' characteristics of 76 patients for Ampliseq

Recently, we reported data from 397 patients who received NAC regarding the evaluation of the clinicopathologic feature of patients with early failure as well as pCR. These data were from the electronic database of the Department of Medicine at the Samsung Medical Center and from retrospectively reviewed records of 433 patients who were diagnosed with histologically confirmed invasive breast cancer and received NAC at the Samsung Medical Center from January 2005 to December 2011 [9]. Among these populations, 76 patients who had available tissue for next generation sequencing (NGS) and nCounter assay were included in this analysis (Figure 1).

### Clinicopathological characteristics of the 76 patients with NAC

The median age at diagnosis of the 76 patients was 46 years (range, 28–63 years; Table 1). Among the 76 patients, 23 patients showed EF, 21 patients had pCR, and the remaining 32 patients were regarded as controls. The subtype distributions was as follows: patients with EF were more likely to have HER2+ BC (39.1%) or TNBC (47.8%). Patients with HR+ BC (ER+ and/or PgR+ and HER2−) had no pCR, and HER2+ patients represented 57.2% of the pCR group. TNBC was present in 42.9% of the patients in the pCR group.

**Table 1:** Patients' characteristics

*N* = 76	Group 1	Group 2	Group 3
	Control	Early Failure	pCR
	*N* = 32 (%)	*N* = 23 (%)	*N* = 21 (%)
Median age (range)	46 (31-63)	45 (28-63)	46 (36-58)
Menopause			
Premenopausal	19 (59.4%)	16 (69.6%)	14 (66.7%)
Peri-Menopausal	3 (9.4%)	0 (0.0%)	0 (0.0%)
Postmenopausal	6 (18.8%)	2 (8.7%)	5 (23.8%)
Unknown	4 (12.5%)	5 (21.7%)	2 (9.5%)
Proportion of IDC	30 (93.8%)	22 (95.7%)	21 (100%)
Subtype			
HR+, HER2−	16 (50.0%)	3 (13.1%)	0 (0.0%)
HR+, HER2+	3 (9.4%)	1 (4.4%)	6 (28.6%)
HER2+, HR−	5 (15.6%)	8 (34.8%)	6 (28.6%)
TN (HR−, HER2−)	8 (25.0%)	11 (47.8%)	9 (42.9%)
Tumour status			
cT1	1 (3.1%)	1 (4.4%)	3 (14.3%)
cT2	14 (43.8 %)	7 (30.4%)	9 (42.9%)
cT3	13 (40.6%)	11 (47.8%)	8 (38.1%)
cT4	4 (12.5%)	4 (17.4%)	1 (4.8%)
Nodal status			
cN1	1 (3.1%)	4 (17.4%)	5 (23.8%)
cN2	22 (68.8%)	10 (43.5%)	12 (57.2%)
cN3	9 (28.1%)	9 (39.1%)	4 (19.1%)
Nuclear grade			
I	3 (9.4%)	0 (0.0%)	0 (0.0%)
II	7 (21.9%)	3 (13.1%)	3 (14.3%)
III	20 (62.5%)	14 (60.9%)	3 (14.3%)
Unknown	2 (6.3%)	6 (26.1%)	15 (71.4%)
Pathologic Stage			
0	0 (0.0%)	0 (0.0%)	21 (100.0%)
I	2 (6.3%)	2 (8.7%)	0 (0.0%)
II	13 (40.6%)	6 (26.1%)	0 (0.0%)
III	16 (50.0%)	10 (43.5%)	0 (0.0%)
Unknown	1 (3.1%)	5 (21.7%)	0 (0.0%)

### Next generation sequencing using Ampliseq with > 1% for mutations with low allele fraction

**Figure 2:**
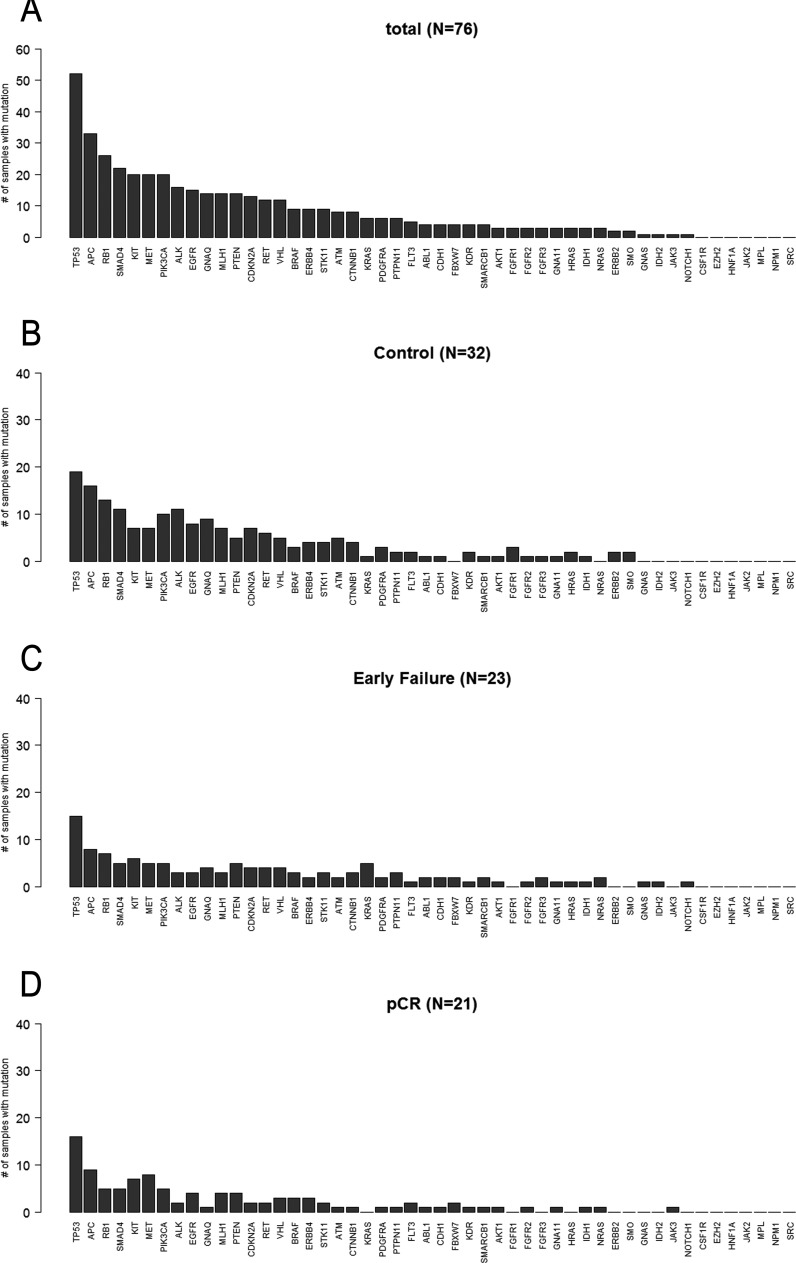
Frequency of mutations in 76 patients for Ampliseq (MAF>0.01) **A.** Total patients (*n* = 76). **B.** Control (*n* = 32). **C.** Early failure (*n* = 23). **D.** pCR (*n* = 21).

Figure 2A shows the number of patients with mutations in 50 genes among 76 patients. Fifty-one of the 76 patients (67.1%) harbored at least one mutation (Figure 2; MAF < 0.01). A total of 532 mutations were detected in samples from 76 patients. Genes in which somatic mutations were detected frequently included TP53 (50 cases, 65.8%), APC (33 cases, 43.4%), RB1 (25 cases, 32.9%), SMAD4 (21 cases, 27.6%), KIT (20 cases, 26.3%), MET, (20 cases, 26.3%), PIK3CA (20 cases, 26.3%), ALK (16 cases, 21.1%), EGFR (15 cases, 19.7%), GNAQ (14 cases, 18.4%), MLH1 (14 cases, 18.4%), PTEN (14 cases, 18.4%), CDKN2A (13 cases, 17.1%), RET (10 cases, 15.8%), and VHL (10 cases, 15.8%), as shown in Figure 2A. Figure 2B-2D shows the number of samples and mutation proportions among the three groups, respectively.

**Figure 3:**
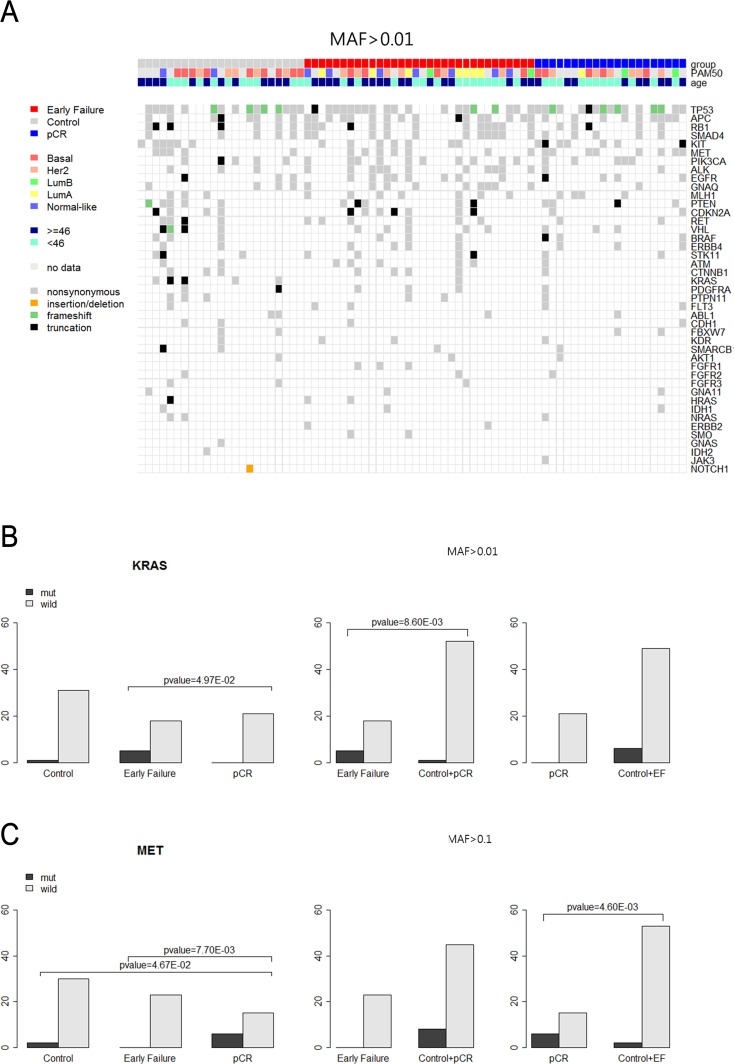
**A.** Heatmap of the mutations found in 76 patients (MAF>0.01). **B.** KAS mutation among three patients' groups (MAF >0.01). **C.** MET mutation among three patients' groups (MAF >0.1).

Figure 3A depicts the heat map of the mutations detected in the 76 patients. Frameshift mutations in the TP53 gene were observed more frequently in patients with pCR than in those with EF (23.8% *vs.* 17.4%). Most of the mutations were nonsynonymous.

KRAS alterations were found in six cases (7.9%) among the 76 samples: five of those were detected in patients with EF. However, there were no KRAS mutations in patients with pCR (Table 2A, 2B; Figure 3B).

**Table 2A:** The mutation of KRAS gene among three groups (MAF>0.01)

	Gene	Group1_wild	Group1_mut	Group2_wild	Group2_mut	*p* value	Group1.ratio	Group2.ratio
Early Failure vs. pCR	KRAS	18	5	21	0	0.0497	0.2174	0
Early Failure vs. Control	KRAS	31	1	18	5	0.8348	0.0312	0.2174
Early Failure vs. pCR + Control	KRAS	18	5	52	1	0.0086	0.2174	0.0189

**Table 2B:** The mutation of KRAS gene: significant at protein level (MAF>0.01)

	Gene	Group1_mut	Group2_mut	Group1_wild	Group2_wild	*p* value
Early Failure vs. pCR	KRAS p.Gly12Val, p.Gly12Ser, p.Gly13Asp	4	0	19	53	0.006902
Early Failure vs. Control + pCR	KRAS p.Gly12Val, p.Gly12Ser	3	0	20	53	0.025192
Early Failure vs. Control	KRAS p.Gly12Val, p.Gly12Ser, p.Gly13Asp	0	4	32	19	0.025964

### > 10% variants for polymorphism

To seek polymorphisms, variants were collected and are shown in Supplementary Figures 1A, 1B, 1C, 1D and 2. Met alterations were significantly more frequent in patients with pCR than they were in those with EF (Table 2C, 2D; Figure 3C).

**Table 2C:** The mutation of MET gene among three groups (MAF>0.1)

	Gene	Group1_wild	Group1_mut	Group2_wild	Group2_mut	*p* value	Group1.ratio	Group2.ratio
pCR vs. Early Failure	MET	23	0	15	6	0.0077	0	0.2857
pCR vs. control	MET	30	2	15	6	0.0467	0.0625	0.2857
pCR vs. Control + Early Failure	MET	53	2	15	6	0.0046	0.0364	0.2857

**Table 2D:** The mutation of MET gene: significant at protein level (MAF>0.1)

	Gene	Group1_mut	Group2_mut	Group1_wild	Group2_wild	*p* value
pCR vs. Control + Early Failure	MET p.Asn375Ser	5	2	16	53	0.015241
pCR vs. Early Failure	MET p.Asn375Ser	0	5	23	16	0.018737

### nCounter assay using NanoString including PAM50 and IHC (Table 3, Figure 4)

**Figure 4:**
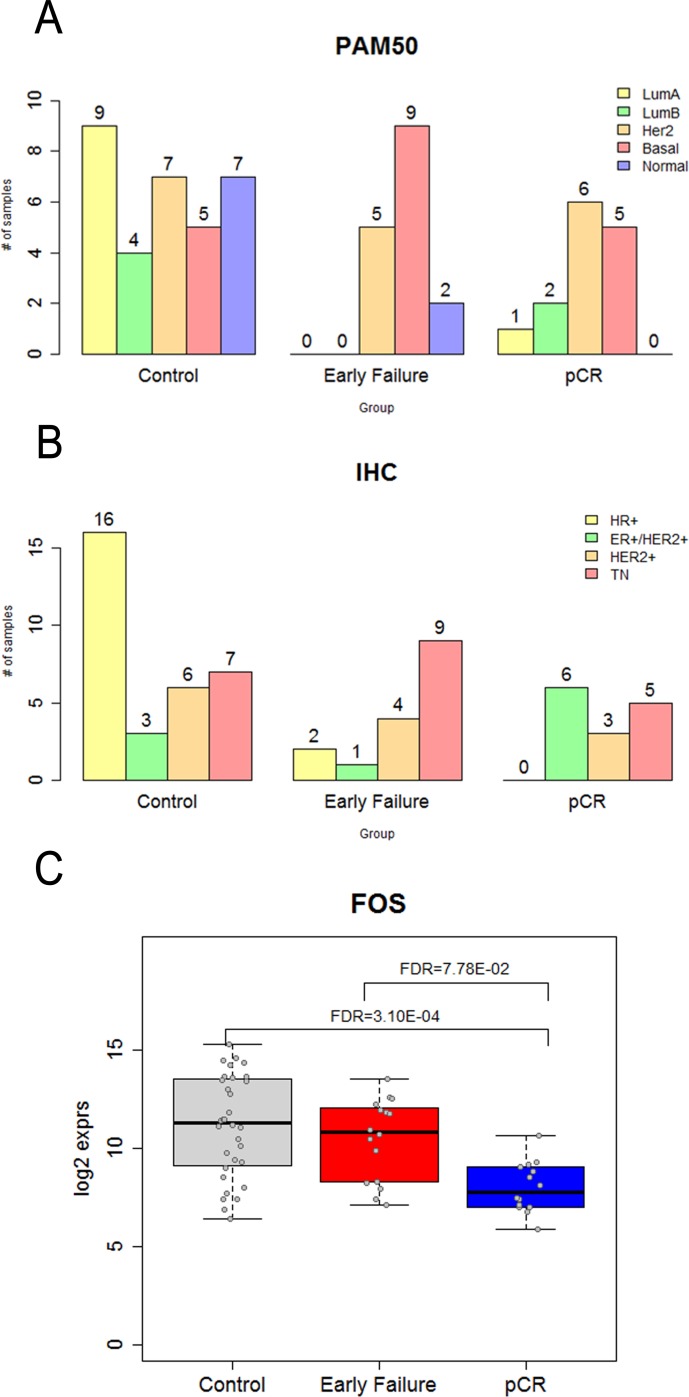
nCounter assay of 62 patients who were available tissue for RNA analysis **A.** PAM50 genes. **B.** Immunohistochemistiry. **C.** FOS gene expression among three groups.

Figure 4A shows the distribution of the intrinsic subtypes classified by PAM50 using the NanoString nCounter assay. This analysis was performed in 62 patients who were available for further nCounter assay using RNA extracts after NGS. In contrast with the control group, the HER2-enriched and basal-like subtypes were composed of mainly of patients with EF and pCR. Compared with the pCR group, the basal-like subtype was found predominantly in the EF group. The IHC distribution of the four subtypes (ER+/HER2-, ER+/HER2+, ER-/HER2+, and triple negative) among the three patient groups receiving NAC (control, EF, and pCR) ia shown in Figure 4A and 4B. The heat map of PAM50 genes detected among the three groups is shown in Supplementary Figure 3.

An expression analysis of 257 genes among the three groups showed that FOS gene expression was significantly higher in patients with EF than it was in those with pCR (Figure 4C). Table 3 shows the list of genes that exhibitedsignificant fold changes among the groups of patients. In addition, the heat map of fold changes in the 257 genes among the three groups is shown in Supplementary Figure 4.

**Table 3:** nCounter assay among three patients' groups from neoadjuvant chemotherapy

Gene	Early Failure	pCR	*p* value	FDR	diff
BMP2	4.055	1.835	5.66.E-04	7.78.E-02	−2.429
FOS	10.475	8.045	6.06.E-04	7.78.E-02	−2.220

## DISCUSSION

Paradoxically, our results showed that the distribution of intrinsic subtypes, as assessed using IHC, between the patients with pCR or EF appeared to be similar even though they had a completely different prognosis. In addition, the PAM50 gene set confirmed this similarity between pCR or EF. Most of the patients with pCR or EF were TNBC and HER2+ subtypes. This represents the extreme tumor heterogeneity of breast cancer, especially in patients with TNBC and HER2+ tumors. Obviously, characterizing pCR is of immense importance and has clinical implications for patients with LABC. For the same reasons, characterizing EF is crucial because of its dismal prognosis in spite of its curable clinical setting, as described in our previous report [9]. Thus, the development of useful predictors for prognostic evaluation or for predicting chemotherapeutic response is urgently needed and will have significant clinical implications.

Therefore, proper treatment strategies for patients with extreme different prognosis could be differentiated after the characterization of pCR and EF in advance using multi-omics on pre-chemotherapy biopsy tissues.

Our results showed that KRAS mutation was enriched in patients with EF compared with pCR and pCR plus control (21.7% *vs.* 0%, 21.7% *vs.* 1.8%) (Table 3A; Figure 3A, 3B). Patients with KRAS codon 12 and 13 mutations seemed to present a worse prognosis with chemotherapy refractoriness and aggressive clinical course, in spite of the curative clinical setting (Table 3A, 3B). The proportion of EF detected in this study was classified as a deeply peculiar clinical setting with significant implications. This result was compatible with another recent study that reported possible prognostic and predictive significance of KRAS alteration together with MYC mutation [10], which was not included in this panel (Supplementary Table 1). This marker may help the clinical stratification of NAC in patients with BC. The predominance of frameshift mutations of TP53 in patients with pCR is compatible with a recent mutational analysis of patients with NAC (Figure 3A) [11].

The other significant finding of this study was the presence of MET gene alterations in patients with pCR (Table 3C, 3D; Figure 3C). Table 3C and 3D showed that MET gene alterations were driven mainly in patients with pCR. No patients with EF had MET gene alterations (MAF > 0.1). In fact, this alteration was found while searching for polymorphisms that contribute to responsiveness to chemotherapy. This alteration contributes to the loss of c-MET affinity to its ligand, HGF, which has been identified as a phantom ligand of MET [12-14]. Lung cancer cells expressing this mutation have been reported to be less sensitive to c-MET inhibition by SU11274. This mutation has been further characterized as a polymorphism because of its increased frequency within general population and its lack of transforming abilities [15, 16]. These findings are supported by recent reports that the HGF/c-MET axis drives cancer aggressiveness [12, 17]. Interestingly, PAM50 analysis using surrogate IHC subtyping showed an even distribution of each subtype between EF and pCR: HER2-enriched and basal-like subtypes were distributed between EF and pCR evenly (Figure 4A, 4B). c-MET mutation may be a plausible explanation for this paradox. MET polymorphism in tumors of the HER2-enriched and basal-like subtype may contribute to responsiveness or refractoriness to NAC even in the same intrinsic subtypes. Moreover, FOS expression appeared to be higher in patients with EF; however, this finding warrants validation in future research.

## CONCLUSION

KRAS gene mutation and c-MET gene polymorphism were associated with EF and pCR in this analysis. Our results support the contention that targeted sequencing using a cancer panel may function to identify actionable targets that are associated with responsiveness or refractoriness to NAC among patients with LABC.

## MATERIALS AND METHODS

### Patients

Seventy-six patients among 397 with LABC (cT2-4N0-3) for whom a preoperative FFPE tumor block was available for NGS were included in this analysis, excluding 22 patients whose fresh-frozen paraffin-embedded (FFPE) blocks were not qualified for AmpliSeq. pCR was defined as the absence of residual tumor both in breast and axillary lymph nodes. The presence of ductal carcinoma in situ was included in pCR. EF was defined as the development of an inoperable state caused by locoregional and/or systemic progression during NAC, or relapse after curative surgery within 1 year after the initiation of NAC. Patients who developed recurrence after 1 year from the start of NAC or exhibited no failure during the follow-up period were defined as controls in this study. Thus, our cohort was composed of three groups: pCR, EF, and control. The clinicopathological characteristics and disease courses of the patients whose disease progressed within 1 year of receiving neoadjuvant chemotherapy were analyzed, for comparison with the other patients. A total of 76 patients with LABC who received NAC and had an available preoperative tumor tissue were included in this analysis. The institutional review board of Samsung Medical Center, Seoul, Korea approved our study protocol (SMC 2013-12-155).

### Immunohistochemical staining

Two experienced pathologists reviewed all pathology specimens to determine the following tumor characteristics: histological and nuclear grades, primary tumor size, presence of lymphovascular invasion, multiplicity, and IHC staining for ER, PgR, and HER2. ER and PgR positivity were defined using Allred scores ranging from 3 to 8 based on IHC using antibodies to the ER (Immunotech, Marseille, France) and PgR (Novocastra Laboratories Ltd., Newcastle upon Tyne, UK). HER2 status was evaluated using a specific antibody (Dako, Glostrop, Denmark) and/or fluorescence in situ hybridization (FISH). Grades 0 and 1 for HER2, as assessed by IHC, were defined as a negative result, and grade 3 was defined as a positive result. Amplification of HER2 was confirmed by FISH if HER2 was rated as 2+ by IHC. Ki67 IHC analyses were evaluated by both independent semiquantitative and quantitative methods (Dako). “Triple negativity” was defined as a lack of expression of ER, PgR, and HER2. Our pathologists also reviewed all core biopsies from referring institutions, including IHC performed at the time of the initial referral, and findings for all surgical specimens, without knowledge of the NanoString results or the treatment outcome. The institutional review board of the Samsung Medical Center, Seoul, Korea approved our study protocol for informed consent waiver and the use of archival tissues with retrospective clinical data.

### DNA extraction

Tissue sample with tumor cell percentage with more than 75% (from 4 mm unstained sections) were dissected under a microscope by comparison with an H&E-stained slide, and genomic DNA was extracted from 76 patients with LABC using a Qiagen DNA FFPE Tissue Kit (Qiagen, Hilden, Germany) according to the manufacturer's instructions. After extraction, we measured DNA concentration as well as 260/280 and 260/230 nm ratios using a spectrophotometer (ND1000; NanoDrop Technologies, Thermo Fisher Scientific, Waltham, MA, USA). Each sample was then quantified using a Qubit fluorometer (Life Technologies, Carlsbad, CA, USA). Genomic DNA with a quantity > 10 ng, as measured on the Qubit fluorometer, was subjected to library preparation.

### RNA extraction

Areas containing representative invasive breast carcinoma were outlined on the slide. Total RNA was extracted from 2-4 sections of 4-μm thick, FFPE sections. Nontumor elements were removed by manual microdissection before transfer to the extraction tube, guided by hematoxylin and eosin stained slides. Then, total RNA was extracted using the High Pure RNA Paraffin kit (Roche Diagnostics, Mannheim, Germany). RNA yield and purity were assessed using a NanoDrop ND-1000 Spectrophotometer (NanoDrop Technologies, Rockland, DE, USA). One sample with less than 50 ng/uL of total RNA, even after concentration using a SpeedVac concentrator (Thermo Scientific™, Waltham, MA, USA) was excluded from downstream analysis, because 200 ng of input RNA in a volume of 5 uL volume needed to be hybridized with 20 uL of probe set master mix.

### NanoString^®^ nCounter Assay using 250 genes probe including PAM50 genes

Gene expression was measured on the NanoString nCounter Analysis System (NanoString Technologies, Seattle, WA, USA). The system measures the relative abundance of each mRNA transcript of interest using a multiplexed hybridization assay and digital readouts of fluorescent bar-coded probes that are hybridized to each transcript [18]. An nCounter CodeSet (NanoString Technologies) containing a biotinylated capture probe for 252 target genes, including 50 PAM50 genes and five housekeeping genes (Supplementary Table 2) and reporter probes attached to color-barcode tags-according to the nCounter™ code-set design-was hybridized in solution to 200 ng of total RNA for 18 h at 65 °C, according to the manufacturer's instructions. Hybridized samples were loaded onto the nCounter Prep Station for posthybridization processing. On the deck of the Prep Station, hybridized samples were purified and immobilized in a sample cartridge for data collection, followed by quantification of the target mRNA in each sample using the nCounter Digital Analyzer. Quantified expression data were analyzed using NanoString's nSolver Analysis Software. After performing image quality control using a predefined cutoff value, we excluded the outlier samples using a normalization factor based on the sum of positive control counts greater than threefold. The counts of the probes were then normalized using the geometric mean of the five housekeeping genes and log2-transformed for further analysis. In total, 62 patients with LABC and 252 genes were used in the statistical analyses.

### Next generation sequencing (NGS) using Ion torrent ampliseq cancer panel v2

Using the Ion Torrent Personal Genome Machine (Ion PGM, Life Technologies, Carlsbad, CA, USA) Cancer Panel v2 (Supplementary Table 1) after DNA isolation from FFPE samples, we sequenced 2,855 loci from 50 cancer-related genes to identify genetic mutations in 76 BC patients who received NAC for LABC and had available preoperative tumor tissue. Libraries were constructed using the Ion AmpliSeq Panels pool (Life Technologies) and 10 ng of DNA sample per pool. The amplicons were then ligated to Ion Xpress Barcode Adapters and purified. Next, multiplexed bar-coded libraries were enriched by clonal amplification using emulsion PCR on Ion Sphere particles (Ion PGM Template OT2 200 Kit, Life Technologies) and loaded onto an Ion 316 Chip. Massively parallel sequencing was carried out on the Ion PGM using the Ion PGM Sequencing 200 Kit v2. The Ion AmpliSeq Cancer Hotspot Panel v2 (www.lifetechnologies.com) covered hot-spot regions of 50 oncogenes and tumor suppressor genes.

The primary filtering process was carried out using the Torrent Suite v3.6.0 and the Ion Torrent Variant Caller v3.6 software. The pipeline includes signaling processing, base calling, quality score assignment, adapter trimming, read alignment to human genome 19 references, mapping QC, coverage analysis, and variant calling. For variant detection, a minimum coverage of 100 reads must be achieved, and at least 5% of mutant reads were selected for variants. Variant calls were further analyzed using the ANNOVAR, which included variant filtering and annotation using the COSMIC database, dbSNP build 137, and amino acid change information.

### Bioinformatic and statistical analysis for ampliseq and nCounter assay

Variant calls from Ion AmpliSeq were further evaluated to reduce potential false-positives. We considered coverage ( > 100) and quality score ( > 30) as filtering criteria. In addition, a minimum threshold of mutant allele fraction (MAF) was taken into account for convincing variants as real: > 1% for mutations with a low allele fraction and > 10% for polymorphisms. For the statistical analysis of final variants, read alignments were manually investigated using the Integrative Genomic Viewer (http://www.broadinstitute.org/igv/). We also discarded the Korean-specific germ-line variants rs1042522 in TP53 and rs1870377 in KDR. Among the variants that satisfied the filtering criteria described above, variants causing amino acid changes and frameshifts were finally chosen for statistical analysis. Fisher's exact test was used for the analysis of mutations and polymorphic variants separately, to discover variants that were enriched in the patients with a favorable outcome. *P*-values < 0.05 were considered significantly different.

For gene expression data from the NanoString nCounter assay, filtering of samples using quality control (QC) criteria was performed according to the manufacturer's recommendations. Raw counts of QC-passed samples were normalized using five reference genes as internal controls (GUSB, PUM1, TBP, TFRC, and TUBB). The QC and normalization mentioned above were performed using the nSolver Analysis Software v2.0 (NanoString Technologies) [19]. Data were log2-transformed and used for further analysis. To compare normalized expression values between groups classified according to clinical outcomes, a t test was used. P-values were adjusted using the FDR correction for multiple comparisons [20]. FDRs less than 0.1 were considered as significantly different.

Intrinsic subtypes classification was performed by using the PAM50 predictor, as described in Parker et al. [21]. To obtain more consistent results, we merged microarray expression data of TCGA breast cancers with our NanoString data after adjusting for batch effects using ComBat algorithm [22], and applied nearest PAM50 centroid algorithm Bioclassifier to predict PAM50 subtypes [21]. All statistical tests, plots and PAM50 subtype prediction were conducted using R version 3.0.2 (http://www.R-project.org/).

### REMARK guidelines

In reporting our study, we have adhered to the guidelines of an important methodological paper from 2005 entitled “Reporting recommendations for tumor marker prognostic studies (REMARK guidelines).” [23, 24]. To decrease any potential bias arising in a review of the medical records, we included ‘Patient Cohort’ analysis to fulfill these criteria (Figure 1).

## SUPPLEMENTARY MATERIAL FIGURES AND TABLES




