# Supplementary material for: Molecular characterization of patients with pathologic complete response or early failure after neoadjuvant chemotherapy for locally advanced breast cancer using next generation sequencing and nCounter assay
Source: Oncotarget. 2015 May 12;6(27):24499–510. doi: 10.18632/oncotarget.4119 (PMC4695201; doi:10.18632/oncotarget.4119)
Supplement: Supplementary file 1 [file oncotarget-06-24499-s001.pdf]

Molecular characterization of patients with pathologic complete response or early failure after neoadjuvant chemotherapy for locally advanced breast cancer using next generation sequencing and nCounter assay

Supplementary Material

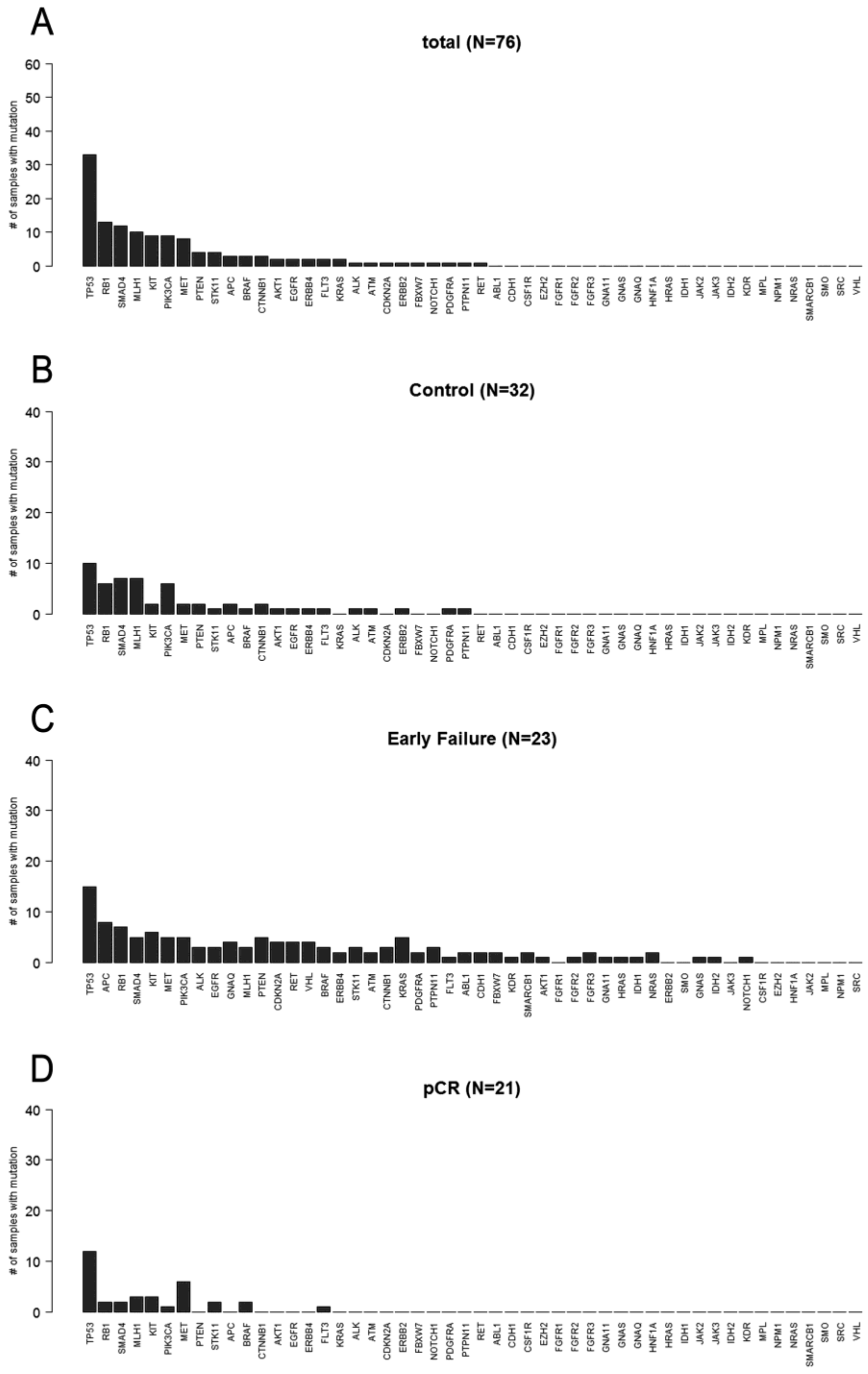

Supplementary Figure 1: Frequency of mutations in 76 patients for Ampliseq (MAF>0.1). **(A)** Total patients (*n* = 76). **(B)** Control (*n* = 32). **(C)** Early failure (*n* = 23). **(D)** pCR (*n* = 21).

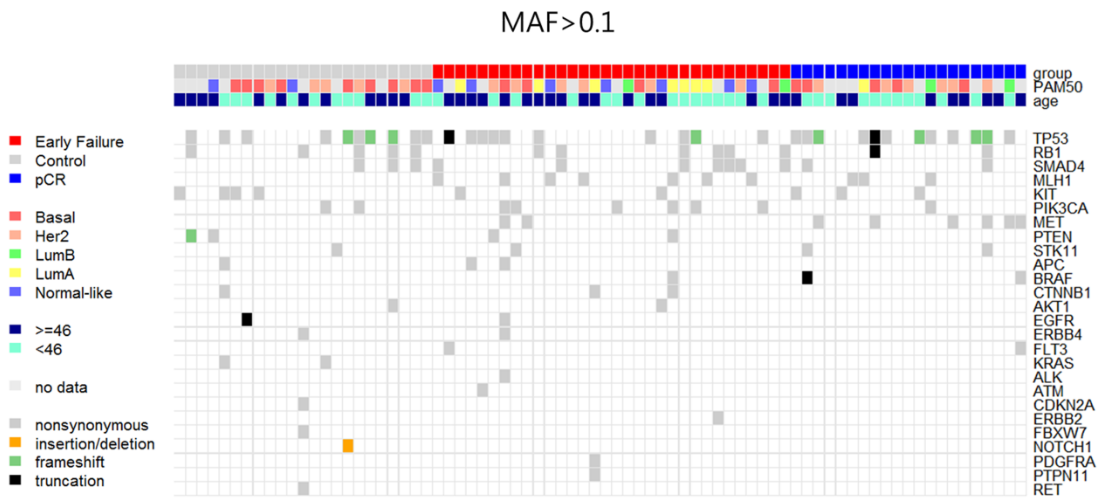

Supplementary Figure 2: Heatmap of the mutations found in 76 patients (MAF>0.1).

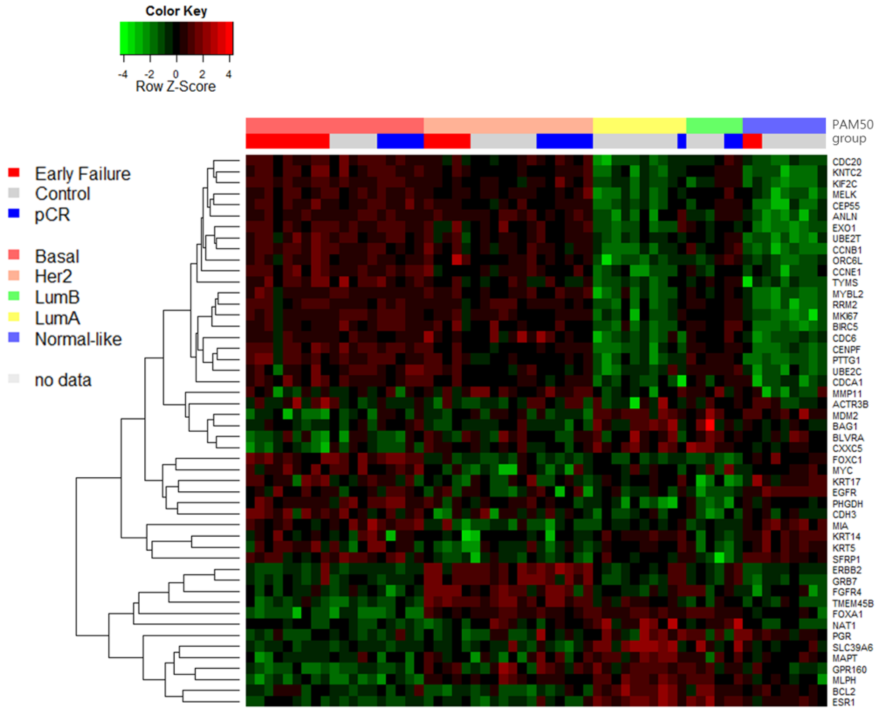

Supplementary Figure 3: Heatmap of PAM50 genes in nCounter assay.

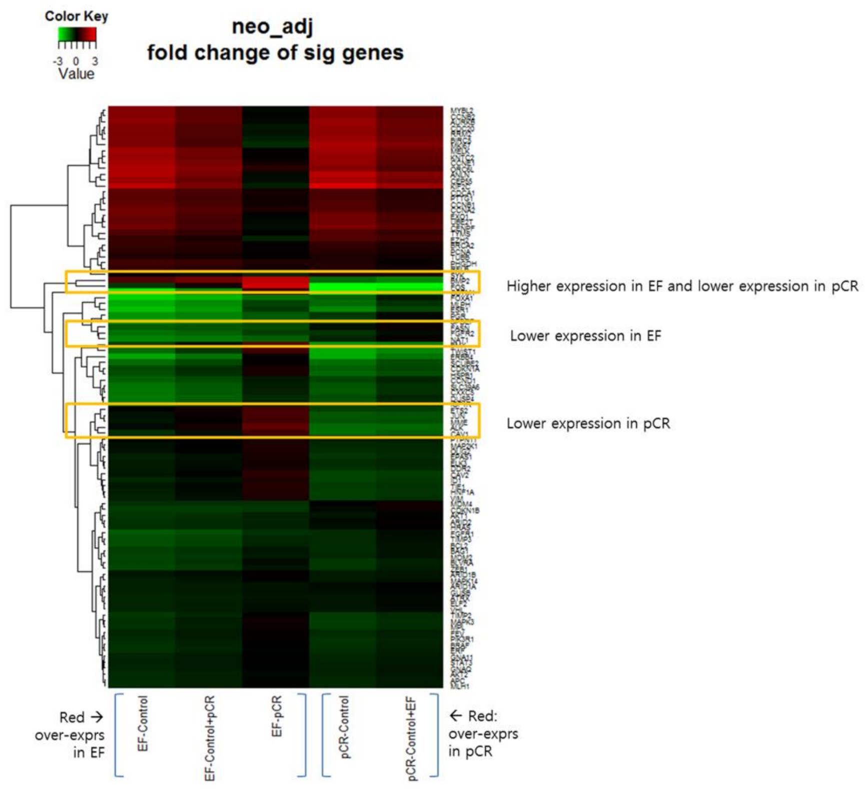

Supplementary Figure 4: Heatmap of fold changes among three groups in nCounter assay utilizing 257 genes.

Supplementary Table 1: The gene list for the Ion Torrent AmpliSeq Cancer Panel

Supplementary Table 2: 257 nCounter gene list including 50 PAM50 genes and 5 reference genes
